# Assessment of Health Behaviours and Satisfaction with Life among Catholic Priests in Poland

**DOI:** 10.1007/s10943-023-01736-4

**Published:** 2023-01-16

**Authors:** Krzysztof Kalita, Justyna Leszczak, Ewelina Czenczek-Lewandowska, Artur Mazur

**Affiliations:** 1 BBIAS Office for Statistical Research and Analysis Rzeszów, Ul. Malownicza 36, 35-304 Rzeszów, Poland; 2 Institute of Health Sciences, Medical College, University of Rzeszów, Al. Rejtana 16C, 35-959 Rzeszów, Poland; 3 Institute of Medical Sciences, Medical College, University of Rzeszów, Al. Rejtana 16C, 35-959 Rzeszów, Poland

**Keywords:** Health behaviours, Catholic priests, Priests, Quality of life, Public health

## Abstract

The aim of the study was to assess the relationship between the health behaviours of diocesan priests in Poland and their level of life satisfaction. The specific goal was to determine the factors that most affect their quality of health (internal health control, the influence of others or coincidence) and life satisfaction (positive mental attitude, preventive behaviour, eating habits, health practices). The study involved 250 diocesan priests from the Podkarpackie region who took part in the research by completing an anonymous questionnaire. The questions were mainly based on tools for assessing health behaviours, namely the HBI—health behaviour inventory, MHLC—multidimensional scale of health locus of control and the SWLS—satisfaction with life scale. The general index of health behaviour of clergymen on the HBI scale was 77.12 ± 16.20 (Me = 78), showing a moderate level on the sten scale. On the MHLC health locus of control scale, the respondents believed that their health depended most on internal control: 25.27 ± 5.10 and the influence of others: 23.13 ± 5.57, are of utmost importance for their health. To a lesser extent they believed that health was dependent on chance: 17.60 ± 5.95. The SWLS satisfaction with life index was 22.51 ± 5.43 (Me = 23), also within the moderate range of the sten scale. The factors most closely related to the assessment of life satisfaction were positive mental attitudes and the overall HBI index. The health behaviours of priests measured by the HBI scale and the level of satisfaction with the life of SWLS clergymen are within a moderate range. On the MHLC scale, priests likewise believe that their health depends on themselves and then on others. They are clearly less likely to indicate accidental causes.

## Introduction

Clergymen as a professional group, like the rest of society, have various health problems (Mook, 2019). This work was aimed at assessing the health behaviour of Catholic diocesan priests in south-eastern Poland and determining the level of life satisfaction in this professional group. Knowledge in this area is necessary to understand what health behaviours characterize priests and to what extent they are satisfied with their lives, but above all, it indicates to what extent priests can be a good example of promoting proper habits of caring for health among the faithful.

Understanding and shaping the health practices of aspiring clergymen can be of particular importance, with cascading effects, as clergy are important role models for their congregants (Anshel & Smith, 2014). Recent research suggests that seminary students who apply a religious framework to their physical health—and who, as a result, have positive intentions to implement and maintain healthy behaviours—often report that they are unable to live up to their aspirations, especially when faced with barriers to health practices that are created by the seminary programme itself (Johnston et al., 2022).

From the very beginning, man has been striving for a certain ideal which is determined by: beauty, full supply of vitality and impeccable health (Yarosh, 2019; Zaidel et al., 2005). For health is the element of human life without which original, unforced joy is lost (Steptoe, 2019). In a sense, man inscribes it into his consciousness as an integral part of the human body. It is health that protects against the experience of pain and suffering, for example it is a guarantee of a fully functional body (Jura & Kozak, 2016; Witard & Ball, 2018).

Health itself often becomes the object of interest only when it worsens or is completely lost. People then try to analyse their previous behaviour, look for the mistakes they have made, wonder if they could have prevented it and what changes to make in their behaviour and lifestyle to limit or stop the destruction of their health state (Tomljenović, 2014; Witard & Ball, 2018).

Classifications of people’s health behaviours take into account, adequate nutrition, physical activity, oral hygiene, hygiene of the environment (home, workplace) (Chevance et al., 2021; Havigerová et al., 2018). It is also necessary to completely eliminate the consumption/use of illicit drugs, and to comply with the relevant safety rules in traffic (not exposing oneself and other road users to the risk of loss of health or life) (Card et al., 2018; Helle et al., 2019; Kramer, 2020; Laberge et al., 2021; Park et al., 2019; Smith et al., 2019).

Health behaviours of various professional groups are a frequent topic analysed in the literature, but priests are the subject of few studies in the field of medicine (Miron et al., 2019; Mirpuri et al., 2021; Ras et al., 2021; Temam et al., 2021; Zwingmann et al., 2011). The health condition of priests and their health behaviour can be significant, because in the context of health, similarly to doctors, they enjoy great social trust and their behaviour can set an example to be followed by society (Proeschold-Bell et al., 2017).

The latest scientific reports show the commitment of religious leaders, which was noticed during the Covid-19 pandemic, when health became a particularly important value for the population. Society has identified religious leaders as key stakeholders in community engagement efforts, including disease prevention (Baruth et al., 2013; Wijesinghe et al., 2022). Undoubtedly, an important aspect is the level of people's religiosity, which differs depending on the place of residence.

ESS data show that the declared religiosity in European countries fell sharply between 2018 and 2002, among others in Ireland (from 83 to 68%), Hungary (from 63 to 50%), Spain (from 78 to 67%), Portugal (from 86 to 76%), Germany, (from 61 to 57%) and Ukraine (from 76 to 72%) (Mentus et al., 2020). There are also countries where religiosity has remained at the current level (Croatia, Slovakia), or is even increasing: Slovenia (from 51 to 57%), Lithuania (83–90%), Austria (71–76%), France (49–53%) and Italy (77–79%) (Bullivant, 2018). The outbreak of the Covid-19 pandemic was a key event in a rise in religiosity in the world. Scientific reports describe that an increase in religiosity occurred in a group of young people, which could be caused by fear and anxiety about the pandemic (Bentzen, 2021; Krok et al., 2021; Social & Demographic Trends, 2020).

So far, numerous positive links have been found between deep spirituality and mental well-being and coping with life. However, there are no reports on the extent to which priests themselves care for their health (Lau, 2018). The position of the clergy in society is respected by people.

Priests show great self-discipline and commitment in the sphere of spirituality, therefore it may be assumed that they are equally disciplined in the context of taking care of their own health (Sawatzky et al., 2005). However, the numerous spiritual duties that require a large commitment of time may, in some way, hinder them in taking care of their health, e.g. preventive examinations or follow-up visits (Avdeenko et al., 2019). A few studies confirm that this population is equally highly exposed to health problems, including stress and occupational burnout (Büssing et al., 2013; Ruiz-Prada et al., 2021), overweight and obesity, (Henein et al., 2021a, 2021b; Proeschold-Bell & LeGrand, 2010), cardiovascular disease (Ike et al., 2007), hypertension (Henein et al., 2021a, 2021b). The priests themselves notice the need for screening tests to prevent hypertension and stress (Moceri & Cox, 2019).

Some reports confirm that the clergy are more physically active than the rest of society, but there are also some places where most of them do not meet the recommended standards (Chiarlitti & Kolen, 2020). Little is known about such health behaviours as nutrition, taking care of oral hygiene, check-ups with specialists, as well as about satisfaction with life in the subjective opinion of clergymen.

## Material and Methods

### Characteristics of the Study Group

The study involved 250 diocesan priests from the Podkarpackie voivodship in Poland (an area with one of the highest levels of religiosity) actively exercising ministry in parishes who are not retired. According to the data of the Central Statistical Office from 2018, the area of Podkarpackie is the most religious region in Poland. 97% of the population declares belonging to the Catholic Church (GUS, 2020).

Nearly 63% of the surveyed priests served as parish priests, while 30% were vicars, 7% were residents and academic chaplains. The mean age of the respondents was 49 years (± 12). Most of the respondents served in parishes located in rural areas, *N* = 62.4%, while the remainder lived in a town/city, *N* = 37.6%. Chronic diseases occurred in over 57% of the respondents and they most often involved the circulatory system, *N* = 24.8%, the digestive system, *N* = 20% and the respiratory system, *N* = 17.2%. These clergy least frequently suffered from chronic diseases of the reproductive system, *N* = 1.2%, nervous system, *N* = 7.6% and urinary system, *N* = 10.8%. The following were excluded from the research: retired priests, monastic priests due to their different principles of functioning (Table 1).

**Table 1:** Characteristics of the study group

Baseline characteristic	*N*	%
Age		
< 30 years	22	8.8
31–40 years	42	16.8
41–50 years	62	24.8
51–60 years	81	32.4
> 60 years	43	17.2
Total	250	100.0
Membership in a diocese		
Archdiocese of Przemyśl	130	52.0
Rzeszów Diocese	120	48.0
Total	250	100.0
Place of ministry		
Town/city	94	37.6
Village	156	62.4
Total	250	100.0
Function performed		
Parish priest	157	62.8
Vicar	75	30.0
Other	18	7.2
Total	250	100.0
Do you have any chronic diseases of the systems listed below?		
Nervous system	19	7.6
Circulatory system	62	24.8
Respiratory system	43	17.2
Digestive system	50	20.0
Urinary system	27	10.8
Reproductive system	3	1.2
I do not have any diseases	107	42.8

### Confidence Levels and Sample Size

The maximum error in the range of ± 5% was adopted for the confidence level *p* = 0.05 (95%), which means that with the known number of diocesan priests serving in the parishes in the area where the research was conducted, at least 250 priests had to be tested. Due to the fact that each priest from the population had an equal probability of being included in the sample statistical inference was used in the analysis.

### Methods

The study was conducted using the survey method. The questionnaires were distributed to over 600 parishes through colporteurs who deliver Catholic newspapers weekly (one or two questionnaires delivered to the parish, depending on the number of priests serving in the area). The cover letter attached to the questionnaires contained a request in which one priest was to be randomly selected to participate in the study.

Each of the respondents gave their informed, written and voluntary consent to participate in the study consisting in filling in the anonymous questionnaire. In accordance with the Helsinki Declaration, study participants were informed about the purpose and course of the study and they agreed to participate in it. Moreover, the consent of the Bioethics Committee at the University of Rzeszow was obtained for the implementation of this research (resolution annex No. 2/02/2019).

Three standardised research tools were used to assess health behaviours, i.e. HBI—health behaviour inventory (Avabil, 2018), MHLC—multidimensional health locus of control scale (Kassianos et al., 2016), SWLS—satisfaction with life scale (Vázquez et al., 2013). SWLS as well as supplementary questions, which concerned in particular sociodemographic variables (for example age, membership in a diocese, place of service (city/village), function performed, the last time preventive examinations were carried out, when was the last visit to a family doctor, occurrence of chronic diseases). All these tools are validated questionnaires designed to evaluate healthy and sick adults.

The selection of the scales was based on the available data on the measurement reliability of research tools, i.e. in the case of HBI the reliability assessed by the test–retest method (after six weeks) was 0.85, for the MHLC scale 0.72, and for the SWLS scale 0.81, which proves the level of satisfactorily allowing the use of tools for scientific and diagnostic purposes. The results of Cronbach's Alpha test among the surveyed priests also indicate a satisfactory degree of reliability: 0.91 for HBI; 0.64 for MHLC—I—internal control version B; 0.68 for MHLC—O—influence of others; 0.67 for MHLC—C—case version B and 0.80 for SWLS (Juczyński, 2012).

The measure of the internal accuracy of the HBI is the "content responsibility" of the statements included in the inventory of forms of health behaviour, determined during several stages of inventory construction. The theoretical validity was assessed by correlating the results of the HBI with the results in tests measuring variables that should be related to health-related behaviours.

In 496 respondents, health practices and preventive behaviours correlated positively with the sense of self-efficacy (Generalized Self-Efficacy Scale—GSES: see part B: 0.31; 0.26, *p* < 0.001), and a positive mental attitude with dispositional optimism (Life Orientation test—LOT: cf. part A: 0.33, *p* < 0.001) and internal locus of health control (Multidimensional Health Locus of Control Scale—MHLC: 0.32, *p*, 0.001). Similarly, the general indicator of health behaviours correlated positively and in a statistically significant manner with the internal locus of health control (MHLC; 0.21, *p* < 0.001), dispositional optimism (LOT; 0.16, *p*, 0.01), self-efficacy (GSES; 0.30, *p* < 0.001) and subjective assessment of one's own health, measured on an analogue scale (0.16, *p* < 0.01) (Juczyński, 2012).

The validity of the Polish version of the scale was assessed by comparing the MHLC results with another clinical study results, as well as with correlations of Levenson's locus of control scales, which measure similar convictions. A large convergence of the discussed scales with their content counterparts in Levenson's scales was found. In a study of 264 adults from a metropolitan environment, it was found that internal control correlates with self-efficacy (0.32; Generalized Self-Efficacy Scale—GSES), self-esteem (0.32; Rosenberg's Self-Esteem Scale—RSES), and with health assessment (0.30; Health Valuation Scale—HVS). This means that the degree of belief that control over one’s own health depends on oneself is associated with self-esteem and effectiveness, and with a high importance attached to health (Juczyński, 2012).

Assessment on the SWLS, satisfaction with life, scale is expressed in the feeling of satisfaction with one's own achievements and conditions. The theoretical validity was estimated by analysing relationships with variables that indirectly reflect the sense of satisfaction with life or have an impact on them.

A positive correlation was found between the level of life satisfaction and self-esteem (0.56; Rosenberg's Self-Value Scale—RSES) and effectiveness (0.38; GSES—see part B) and dispositional optimism (0.45—LOT-R; item part A), carried out in a group of 272 adults. Moreover, the result of the SWLS scale negatively correlated with the intensity of perceived stress (− 0.58; Perceived Stress Scale—PSS) and the control of emotions of anger, depression and anxiety (− 0.18; 0.23 and 0.24, respectively; cf. CECS in part A) (Juczyński, 2012).

### Description of the Tools

*The Health Behaviour Inventory of the HBI* contains 24 statements describing various types of behaviour related to health and is intended for studying healthy and sick adults. The respondent indicates how often he/she has performed the given health-related activities in the last year. In the interpretation of the overall HBI score, the numerical values of the scale are counted, where a higher score indicates a greater propensity towards the declared health behaviours. Additionally, the propensity towards four categories of behaviour is calculated: correct eating habits, preventive behaviours, positive mental attitude and health practices (Juczyński, 2012). The listed behaviours in the inventory are assessed on a five-point Likert scale. Its purpose is to determine the relative strength of various attitudes and views. The HBI tool shows a satisfactory degree of reliability, as evidenced by high coefficients in the Cronbach's Alpha test (Babbie, 2008).

*MHLC—multidimensional health locus of control scale (version B)*, the scale contains 18 statements and beliefs, which, after appropriate conversions, inform about who or what in the respondent’s opinion decides about their health. According to the scale’s key, these are three variables:

 − internal health control (I): control over my own health depends on me;
 − influence of others (O): my own health is the result of the influence of others, especially medical personnel,
 − chance (C): my health condition is determined by chance or other external factors

At the root of the scale is the assumption that an internal health locus of control promotes healthy behaviours, thus being physically active, limiting tobacco and alcohol consumption, controlling weight, etc. The questions in the research tool are built on a six-point ordinal scale where the respondent expresses his attitude to the statements presented from "I strongly disagree" to "I strongly agree" (Juczyński, 2012).

*SWLS* Satisfaction with life scale that allows assessment of the feeling of satisfaction with one's own achievements and conditions. It consists of five statements built on a seven-point ordinal scale (ranging from "I completely disagree" to "I completely agree"), where the respondent assesses to what extent each of them applies to his current life. The higher the total score, which is the sum of the numbers assigned to the statements, the higher the sense of satisfaction with life (Juczyński, 2012).

When interpreting the total scores of both the HBI and SWLS, the properties characterizing the sten scale should be followed. It is a psychological test scale divided into 10 units and normalized so that the population mean is 5.5 and the standard deviation is 2. Sten scores within 1–4 are considered low, and within 7–10 sten as high. Results within 5 and 6 of the sten are considered average (Hornowska, 2019). Table 2 includes the Polish standard values of the HBI and SWLS scales for men, taking into account the Sten scale (Table 2).

**Table 2:** Polish HBI and SWLS standards for men

Raw result of HBI	STEN
24–50	1
51–58	2
59–65	3
66–71	4
72–78	5
79–86	6
87–93	7
94–101	8
102–108	9
109–120	10

Raw result of SWLS	STEN
5–9	1
10–11	2
12–14	3
15–17	4
18–20	5
21–23	6
24–26	7
27–28	8
29–30	9
31–35	10

### Statistical Analysis

Pearson's r and Spearman's rho coefficients were used to assess the degree of correlation between quantitative variables, where the maximum degree of correlation is 1 (for a positive correlation) or − 1 (for a negative correlation). The closer the strength of a relationship approaches 1 or − 1, the stronger the relationship observed (Bedyńska & Cypryańska, 2013).

Models based on linear regression using the stepwise method, which is one of the parametric methods, were also used. The condition for applying linear regression with the stepwise method is meeting the assumptions of, among others the normal distribution of the residuals and their lack of correlation. This method of analysing models is possible when the dependent and independent variables are quantitative, although it is also possible for the predictors (independent variables) to be dichotomous. Statistical analysis was performed using the SPSS 17.0 statistical package and the adopted significance level was *p* ≤ 0.05.

## Results

### Health Behaviours

The general index of health behaviour of clergymen on the HBI scale was 77.12 ± 16.20 (Me = 78). Individual domains that made up the overall health behaviour were in the middle of the STEN scale, although there was a slightly higher value for positive mental attitude (Me = 3.5).

The results of the MHLC Multidimensional Health Locus of Control scale showed that, in a similar way, the subjects were convinced that internal control 25.27 ± 5.10 and the influence of others 23.13 ± 5.57 were of significant importance in terms of their health. On the other hand, health was considered to be less dependent on chance—17.60 ± 5.95.

The SWLS satisfaction with life index was 22.51 ± 5.43 (Me = 23), therefore it falls within the moderate range of the STEN scale (Table 3).

**Table 3:** General indicators of health behaviours, health locus of control and satisfaction with life

	$\overline{x }$	(95% Cl)	Me	SD ±	Min	Max
HBI scale						
Positive mental attitude (1–5 pts)	3.42	3.32–3.51	3.50	0.74	1.50	4.83
Preventive behaviour (1–5 pts)	3.15	3.05–3.25	3.17	0.82	1.00	5.00
Proper eating habits (1–5 pts)	3.17	3.07–3.28	3.17	0.86	1.00	5.00
Health practices (1–5 pts)	3.12	3.02–3.21	3.17	0.75	1.33	4.67
Total HBI (24–120 pts)	77.12	75.10–79.14	78.00	16.20	39.00	114.00
Health locus of control MHLC						
MHLC—I—internal control (6–36 pts)		24.63–25.90	25.00	5.10	11.00	36.00
MHLC—O—influence of others (6–36 pts)		22.43–23.82	23.00	5.57	9.00	36.00
MHLC—C—chance (6–36 pts)		16.86–18.34	17.00	5.95	6.00	34.00
Satisfaction with life SWLS						
SWLS (5–35 pts)	22.51	21.83–25.90	23.00	5.43	6.00	35.00

*Max*—Maximum value, *Me*—median, *Min*—minimum value, *SD*—standard deviation, $\overline{{\varvec{x}} }$—average value, *CI*—confidence interval

Spearman's rho and Pearson's r correlation values were similar. Positive relationships were found between SWLS and HBI and MHLC, most of which were of moderate level. The highest degrees of correlation indicate that with the increase in the values of the scales: positive mental attitude and the general index of HBI, the SWLS values, which indicate satisfaction with life, increased. There was no statistically significant correlation between MHLC—C—chance and SWLS (Table 4).

**Table 4:** Correlations between HBI health behaviours, the MHLC multidimensional health locus of control scale and SWLS satisfaction with life

SWLS correlations (5–35 pts)		Rho Spearman	*r*-Pearson
HBI. Positive mental attitude (6–30 pts)	Correlation coefficient	0.431^**^	0.460^**^
	Significance (two-sided)	0.000	0.000
	*N*	250	250
HBI. Preventive behaviours (6–30 pts)	Correlation coefficient	0.291^**^	0.285^**^
	Significance (two-sided)	0.000	0.000
	*N*	250	250
HBI. Proper eating habits (6–30 pts)	Correlation coefficient	0.259^**^	0.252^**^
	Significance (two-sided)	0.000	0.000
	*N*	250	250
HBI. Health practices (6–30 pts)	Correlation coefficient	.370^**^	.396^**^
	Significance (two-sided)	0.000	0.000
	*N*	250	250
Total HBI (24–120 pts)	Correlation coefficient	0.389^**^	0.402^**^
	Significance (two-sided)	0.000	0.000
	*N*	250	250
MHLC—I—internal control (6–36 pts)	Correlation coefficient	0.324^**^	0.321^**^
	Significance (two-sided)	0.000	0.000
	*N*	250	250
MHLC—O—influence of others (6–36 pts)	Correlation coefficient	0.131^*^	0.117^*^
	Significance (two-sided)	0.038	0.050
	*N*	250	250
MHLC—C—chance (6–36 pts)	Correlation coefficient	0.044	0.052
	Significance (two-sided)	0.491	0.410
	*N*	250	250

*Correlation is significant at the 0.05 level (2-tailed)

**Correlation is significant at the 0.01 level (2-tailed)

The greater the belief among the respondents that the influence of others and internal control determine health, the more the respondents showed better health behaviours in the HBI. The values of Spearman's rho and Pearson's r show a slight but visible correlation between the analysed variables. There is no statistically significant relationship between MHLC—C—chance and the overall HBI index (Table 5).

**Table 5:** Correlations between the MHLC multidimensional health locus of control scale and the HBI general index of health behaviour

Total HBI correlations (24–120 pts)		Rho Spearman	*r*-Pearson
MHLC—I—internal control (6–36 pts)	Correlation coefficient	0.271^**^	0.264^**^
	Significance (two-sided)	0.000	0.000
	*N*	250	250
MHLC—O—influence of others (6–36 pts)	Correlation coefficient	0.343^**^	0.365^**^
	Significance (two-sided)	0.000	0.000
	*N*	250	250
MHLC—C—chance (6–36 pts)	Correlation coefficient	0.066	0.096
	Significance (two-sided)	0.297	0.130
	*N*	250	250

**Correlation is significant at the 0.01 level (2-tailed)

### Models Explaining Health Behaviour and Satisfaction with Life among Priests

With regard to the dependent variable, the level of HBI health behaviours, four predictors were taken into account: SWLS, MHLC—O, alcohol consumption at least once a week and age, which in total account for approximately 33% of the variance of the dependent variable. Standardized regression coefficients have shown that a better level of HBI health behaviours among the priests, in particular, is influenced by greater satisfaction with life (Beta = 0.32, *p* < 0.001), followed by the belief that health depends on the influence of others (Beta = 0.25, *p* < 0.001), no frequent alcohol consumption (Beta = 0.25, *p* < 0.001) and to a lesser extent, older age (Beta = 0.25, *p* = 0.02) (Table 6).

**Table 6:** Values of the coefficients explaining HBI health behaviours

Model	Non-standardized coefficients		Standardized coefficients	*t*	Significance
	B	Standard error	Beta		
(Constant)	12.142	6.074		1.999	0.047
SWLS (5–35 pts)	0.944	0.159	0.316	5.943	0.000
MHLC—I—influence of others (6–36 pts)	0.741	0.157	0.254	4.711	0.000
Consuming alcohol at least once a week*	10.246	2.164	0.255	4.735	0.000
Age	0.168	0.074	0.120	2.273	0.024

*The results of alcohol consumption with a frequency of less than once a week were encoded with a higher value

With regard to the dependent variable SWLS satisfaction with life, two predictors were taken into account: HBI positive mental attitude and MHLC—I internal control, which explain a total of about 25% of the variance of the dependent variable. Standardized regression coefficients showed that better SWLS satisfaction with life among the priests, in particular, is influenced by HBI positive mental attitude (Beta = 0.40, *p* < 0.001), followed by the belief that health is determined by internal control (Beta = 0.21, *p* < 0.001) (Table 7).

**Table 7:** Values of the coefficients explaining SWLS satisfaction with life

Model	Non-standardized coefficients		Standardized coefficients	*t*	Significance
	B	Standard error	Beta		
(Constant)	6.686	1.819		3.675	0.000
Positive mental attitude (1–5 pts)	2.949	0.418	0.402	7.054	0.000
MHLC—I—internal control (6–36 pts)	0.228	0.061	0.214	3.749	0.000

## Discussion

The aim of the study was to evaluate the health behaviour of Catholic diocesan priests in south-eastern Poland and to determine the level of life satisfaction in this professional group. The presented results in this regard allowed for a better understanding of what health behaviours priests are characterized by and to what extent they are satisfied with their lives, but above all, literature suggests that these results could be used to show how much priests might be a good example of the promotion of proper habits of caring for health among the faithful. The obtained results constitute an important supplement to the existing literature, because so far the focus has been most often on the role of priests in caring for the health of the society, while less is known about health behaviours and satisfaction with the life of priests themselves and ultimately on the potential influence these behaviours might have on their parishioners health.

The clergy are a respected social category that are considered by most believers as a model of behaviour, including in the area of caring for their own health. However, too little attention has been paid to the objective examination of priests' health, their health behaviour and satisfaction with life. Our own research showed that the general index of health behaviour in the HBI scale, as well as the SWLS satisfaction with life index of priests were within the moderate range of the STEN scale. When it comes to locating the factors that influence health control, it is internal control and the influence of others that have a significant impact on their health. To a lesser extent, the respondents believed that health depends on chance.

As indicated by the author of the MHLC (Rotter, 1954), the tool is based on the assumption that the internal locus of health control is conducive to pro-health behaviours, i.e. taking up physical activity, reducing smoking and drinking alcohol, controlling body weight or, for example, preventing infections. The research of other authors shows that among adults the sense of the importance of internal control decreases with age, while the importance of the influence on their own health of other people (e.g. doctors) and of chance increases (Juczyński, 2012).

Webb and Chase (2019) draw attention to the important role of professional stress, which may affect priests and have a direct impact on their physical and mental health as well as on health behaviour patterns. The higher the level of occupational stress, the more often priests reported health problems, such as hypertension, diabetes, depression and more time spent sitting and more hours worked per week. Thus, they confirm that both professional stress and numerous health problems are a current problem for priests, regardless of age and professional experience (Webb & Chase, 2019). Similarly, Ruiz-Prada et al. reviewing the available literature, state that workload is more common among younger priests. They also report that stress and burnout are associated with factors such as smoking, alcoholism, obesity, diabetes, cardiovascular disease, anxiety and depression. Strengthening protective factors and minimizing the impact of risk factors would greatly contribute to the improvement of the health of the clergy profession (Ruiz-Prada et al., 2021).

The average level of health behaviour of priests in our own research is considered by some scientists as a result of a religious vocation (Bussing et al., 2009; Dobrakowski et al., 2021). This was the result of, among other things, pilot studies among Christian and Jewish clergy on how their vocation affects their health. A total of 36 clergymen in Pennsylvania aged 54.6 ± 8.44 participated in the interview. During the conducted analysis, it was shown that the majority of the priests (61.1%) believed that their ministry had a negative impact on their health. Factors such as stress, unhealthy eating, lack of physical activity and lack of time for self-care were identified as negative health effects among the clergy. The results confirm that the vocation may increase the risk of negative health effects due to the busy lifestyle where priests largely minister to others and consequently have no time for themselves. Such an assessment applies to all clergymen, regardless of their religious tradition or denominational affiliation.

(Webb et al., 2016). Similarly, Kane emphasizes that although most priests are highly aware of health-promoting behaviours (diet, physical activity, health care), they experience stress, most of which results from high professional demands and loneliness, which they also experience (Kane, 2017).

Chiarlitti and Kolen describe that priests face barriers in terms of time and energy, which result in fewer opportunities to take care of themselves, including participating in regular physical activity. The physical activity barrier can be related to social identities that support or prohibit engaging in physical activity. The main motivations for taking up physical activity were health benefits, and the obstacles were a busy work schedule, lack of knowledge, and poor health preventing participation in physical activity. The authors suggest that physical activity programming should be considered as part of seminary education, in particular as a method of proactively caring for physical and mental health so that priests can cope with the persistent and consistent demands placed on them. Physical activity programmes should include an individualized approach that incorporates personal interests and available resources, while recognizing personal, social and environmental barriers (Chiarlitti & Kolen, 2020). Similar beliefs are presented in research by Johnston et al. who claim that physical health, including planning of actions and coping, should be implemented already in the seminary (Johnston et al., 2022).

Many reports confirm that high religiosity has, above all, positive effects on mental health and the ability to cope with life situations. An important role of religiosity in dealing with mental problems, i.e. mainly depression and schizophrenia, is described by Magliano et al. as well as Tepper et al. These authors confirm that as many as 80% of patients indicated religiosity as a factor helping in coping with mental health (Magliano et al., 2018; Tepper et al., 2001). Heseltine-Carp et al. emphasize the important role of priests in supporting the mental condition of the faithful and call them "frontline mental health workers and gatekeepers to mental health services". However, clergy themselves generally do not receive referrals to mental health professionals, which is an important issue worthy of attention. This proves the necessity of examining the health of priests and encouraging them to pay more attention to their own health, and not only to that of the faithful (Heseltine-Carp & Hoskins, 2020).

Lau, examining 804 priests, showed that a significant percentage of priests (23.4%) had HADS results indicating a possible anxiety problem. Slightly fewer had problems with depression (9.3%). Both anxiety and depression difficulties were associated with an imbalance between the effort put into work and the rewards received at work. Lau in his research showed that among priests there is a higher incidence of mental health problems than among ordinary people.

In our own study on the HBI scale, the highest score was obtained in the field of mental well-being, which in turn was associated with a higher assessment of satisfaction with life. Chourasiya et al. obtained similar results, also noting that among a studied group of priests, the majority (60%) described their mental state as excellent. Despite the fact that the HBI scale did not show a significant problem in terms of mental well-being, monitoring the health of priests is extremely important (Chourasiya et al., 2016).

It is worth emphasizing that despite the good attitudes among the priests, the studied group are also equally exposed to other health problems. Although in numerous studies, priests, usually making a subjective self-assessment of their health condition, most often describe it as good, very good or excellent, when making an objective assessment of health disorders, it turns out that this group is equally exposed to the current problems of public health.

Lindholm describes that over 77% of the priests surveyed were overweight or obese. On the other hand, the greatest barrier to leading a healthy lifestyle is indicated by spending little or no time with family (Lindholm et al., 2016). Baruth et al., (2014), analysing a group of 40 priests, describe that as many as 93% of them were overweight or obese, 68% had hypertension, while 50% had two or more chronic diseases, 35% too high cholesterol, 30% degenerative diseases and 20% diabetes. Pastors took great care regarding check-ups with specialists: 98% had a visit with a specialist along with blood pressure (95%) and blood sugar (88%) measurements during the year. Most of the clergy did not smoke (98%), consumed 3.4 ± 4.0 portions of fruit and vegetables per day, and had a lower than average stress level (2.0 ± 0.7). The priests themselves notice the need for screening tests to prevent hypertension and stress (Moceri & Cox, 2019).

Due to the increasing occurrence of civilization and professional diseases, including among priests, and the level of satisfaction with life, it is necessary to introduce early prophylaxis of these diseases, as well as of chronic diseases. Screening tests are also needed to detect diseases early. It is also important to disseminate knowledge about the positive impact of physical activity on the physical and mental health of a person (including priests). That is why it is important to learn about health behaviours and life satisfaction for early diagnosis of disturbing symptoms that may lead to serious health complications and diseases at a later stage.

### Limitations of This Study

Despite great efforts, the authors of this study did not avoid certain limitations. First, the study was conducted on the basis of the questionnaire method, so the responses of the respondents should be interpreted as subjective.

In the future studies, the analysis of factors that may affect the quality of priests' health should be deepened, including: lifestyle, in which separate issues should be taken into account, such as diet, physical activity, rest, the ability to deal with stress, lack of stimulants and aggression, as well as biological, for example genetic influences, inborn predispositions, features of the immune system, biochemical, physiological and anatomical features of the subject and his family. Secondly, in subsequent studies, objective research tools should be included, for example, to assess the activity—an accelerometer that will test the actual activity of priests over a period of 7 days. An analysis of the availability of medical services would also be worthy of attention.

### Recommendations

Recent research suggests that seminarians may have positive intentions with regards to maintaining optimum health but are often unable to live up to their aspirations due to barriers to health practices that are created by the seminary programme itself (Johnston et al., 2022).

1. Include opportunities for seminarians to establish sound health caring behaviours because there is a cascading effects when these seminarians graduate and become priests and role models for their congregants (Anshel & Smith, 2014).
2. Provide sufficient time for seminarians and priests to incorporate health caring behaviours and their responsibility as role models in their day to day duties.
3. Future research which
  - include: analysis of factors that may affect the quality of priests' including those items listed in the limitations section of this paper,
  - utilizes objective measurements of health,
  - includes seminarians in the study group.

## Conclusions

This work assessed the health behaviour and levels of life satisfaction of Catholic diocesan priests in south-eastern Poland. It filled a gap in the research by focusing on the health behaviours of priests rather than on their role of caring. This study showed that the health behaviours of priests measured by the HBI scale and the level of satisfaction with the life measured by the SWLS were within a moderate range. On the MHLC scale, priests believed that their health depends on themselves and then on others. They are less likely to indicate accidental causes. The factors most closely related to the assessment of life satisfaction were positive mental attitudes and the overall HBI index. Knowledge in this area is necessary because literature indicates priests are role models for their congregations and as such may help to promote proper habits of caring for health among the faithful.
